# Prevalence and Severity of Coronary Artery Calcification Assessed by Low‐Dose Computed Tomography in Individuals With Type 2 Diabetes With and Without Diabetic Foot: A Cross‐Sectional Study

**DOI:** 10.1111/1753-0407.70247

**Published:** 2026-06-17

**Authors:** Feiyan Shi, Yun Gao, Mingxin Bai, Ruixue Feng, Chunjie Ma, Dawei Chen, Chun Wang, Lihong Chen, Sen He, Donge Yan, Chunchao Xia, Xingwu Ran

**Affiliations:** ^1^ Department of Endocrinology and Metabolism, Innovation Research Center for Diabetic Foot, Diabetic Foot Care Center, West China Hospital Sichuan University Chengdu China; ^2^ Center for High Altitude Medicine, West China Hospital Sichuan University Chengdu China; ^3^ Department of Cardiology, West China Hospital Sichuan University Chengdu China; ^4^ Department of Radiology, West China Hospital Sichuan University Chengdu China

**Keywords:** coronary artery calcification, diabetic foot, low‐dose computed tomography, type 2 diabetes mellitus

## Abstract

**Background:**

To investigate whether diabetic foot (DF) is associated with a higher prevalence and severity of coronary artery calcification (CAC) in patients with type 2 diabetes (T2DM) using low‐dose computed tomography (LDCT).

**Methods:**

This cross‐sectional study included 710 patients with DF and 1176 patients with T2DM but without DF who had undergone LDCT recruited in the Diabetic Foot Care Center of West China Hospital, Sichuan University. The severity of CAC was evaluated using a length‐based grading method. The independent association between DF and CAC was evaluated using multivariable logistic regression and propensity score matching (PSM).

**Results:**

The overall prevalence of CAC among individuals with DF was 71.9%, compared to 46.1% in those without DF. DF was independently associated with CAC; an association remained statistically significant after PSM (adjusted OR 1.72; 95% CI [1.28–2.32]; *p* < 0.001). This association was particularly strong in patients with preserved renal function, irrespective of the presence of peripheral artery disease (PAD). In contrast, among individuals with impaired renal function, no significant differences were observed in the prevalence of mild, moderate, or severe CAC between those with DF and those without DF. However, the overall prevalence of CAC across all severity levels was higher in the renal‐impaired group than in those with preserved renal function.

**Conclusions:**

DF is independently associated with the presence and severity of CAC in patients with T2DM, particularly among those with preserved renal function, irrespective of the presence of concomitant PAD. This association is not statistically significant in individuals with impaired renal function.

## Highlights

1. DF is independently associated with a significantly higher prevalence and severity of CAC in T2DM.
2. This association persists in patients with preserved renal function regardless of concomitant PAD, but is not statistically significant in individuals with impaired renal function.
3. Among patients with type 2 diabetes, in addition to those who develop impaired renal function, those who develop DF also warrant special attention for CAC.

## Graphical abstract

Diabetic foot is independently associated with the presence and severity of coronary artery calcification in patients with type 2 diabetes. Patients developing diabetic foot or with impaired renal function may warrant enhanced cardiovascular risk assessment and screening for silent coronary artery disease.

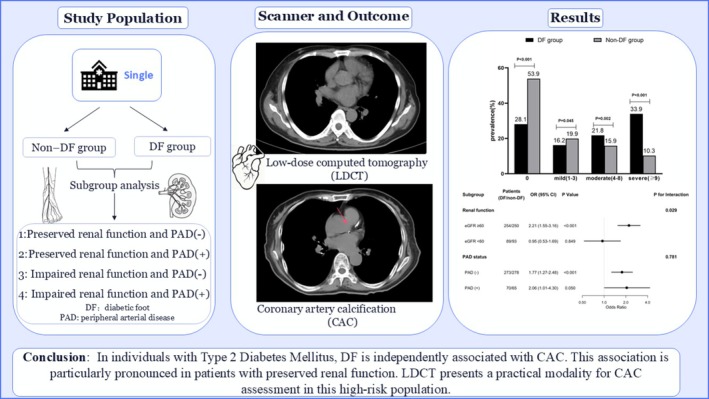

**Abbreviations**
ABI: ankle brachial index
ACR: albumin‐to‐creatinine ratio
AGEs: advanced glycation end products
BMI: body mass index
CAC: coronary artery calcification
CAD: coronary artery disease
CIN: contrast‐induced nephropathy
CTA: computed tomography angiography
CVD: cerebrovascular disease
DBP: diastolic blood pressure
DF: diabetic foot
DM: diabetes mellitus
DPN: diabetic peripheral neuropathy
DPP‐4i: dipeptidyl peptidase‐4 inhibitors
DR: diabetic retinopathy
ECG‐gated CT: electrocardiogram‐gated computed tomography
eGFR: estimated glomerular filtration rate
GLP‐1RA: glucagon‐like peptide‐1 receptor agonists
Hb: hemoglobin
HbA1c: glycated hemoglobin
HBP: hypertension
HDL‐C: high‐density lipoprotein cholesterol
LDCT: low‐dose computed tomography
LDL‐C: low‐density lipoprotein cholesterol
MBP: mean blood pressure
PAD: peripheral arterial disease
PSM: propensity score matching
SBP: systolic blood pressure
SGLT‐2i: sodium‐glucose cotransporter 2 inhibitors
T2DM: type 2 diabetes mellitus
TC: total cholesterol
TG: triglycerides

## 1 Introduction

Diabetic foot (DF) is a severe and incapacitating complication of diabetes mellitus (DM), representing a significant global public health challenge due to its association with heightened morbidity and considerable socioeconomic burden [1]. Individuals with DF face cardiovascular mortality rates 2.22 to 3.27 times higher than those without DF, resulting in a five‐year survival rate on par with or even worse than that observed in certain types of cancer [2, 3]. Despite these alarming statistics, cardiovascular diseases, particularly coronary artery disease (CAD), remain markedly underdiagnosed in this population.

Traditional diagnostic approaches, which largely depend on medical history and cardiac echocardiography, are insufficient in accurately assessing the actual prevalence of CAD.

For instance, observational studies indicate that the reported prevalence of CAD among patients with DF reaches 22.6% when diagnosis is based solely on medical history [4]. The addition of echocardiography as a supplementary diagnostic tool yields only a marginal increase in detection rates, raising the prevalence to 32.4% [5]. In contrast, systematic screening utilizing coronary CT angiography (CTA) reveals a substantially higher prevalence of 63.4%, particularly among patients with ischemic DF [6]. The underdiagnosis of CAD is further exacerbated by the limited applicability of CTA due to the invasive administration of contrast agents and the increased risk of contrast‐induced nephropathy in DF patients, with an incidence rate four times that of non‐DF controls [7]. Therefore, the development of noninvasive methods for early detection of CAD could facilitate timely risk stratification, guide targeted therapeutic interventions, and potentially reduce cardiovascular mortality in this patient population.

Coronary artery calcification (CAC) serves as a biomarker for atherosclerotic burden and cardiovascular risk [8]. The assessment of CAC through coronary artery calcification score (CACS) provides high diagnostic specificity for CAD [9]. The current reference standard for obtaining the CACS is electrocardiogram (ECG)‐gated computed tomography (CT), which employs the Agatston scoring method. However, due to its dependence on specialized equipment and technically complex procedures, its application remains limited in primary healthcare settings, such as county hospitals and community health centers [10, 11, 12]. In contrast, non‐ECG‐gated CT modalities, particularly low‐dose computed tomography (LDCT), have demonstrated strong concordance with ECG‐gated CT in quantifying CAC [13]. Given its widespread use in lung disease screening and evaluation, LDCT offers greater accessibility and lower radiation exposure, making it a viable alternative for CAC assessment in both tertiary hospitals and primary care settings [14, 15]. Furthermore, guidelines from the 2016 Society of Cardiovascular Computed Tomography and the Society of Thoracic Radiology recommend detecting CAC in non‐contrast chest CT scans through visual assessment, thereby helping overcome the temporal constraints associated with ECG‐gated CT and supporting opportunistic early detection of CAD. Consequently, utilizing LDCT for visual CAC assessment facilitates timely and targeted interventions for high‐risk diabetic populations, thereby promoting more efficient resource allocation and alleviating socioeconomic burdens [16].

Although existing clinical evidence suggests that patients with DF tend to exhibit more calcification compared to those without DF [17], there remains a paucity of large‐scale, systematic studies investigating the difference in the prevalence and severity of CAC between these two populations. To address this research gap, we propose a large‐sample clinical study utilizing LDCT to quantitatively assess both the prevalence and severity of coronary calcification in patients with DF relative to non‐DF controls.

## 2 Methods

### 2.1 Study Design and Population

**FIGURE 1:**
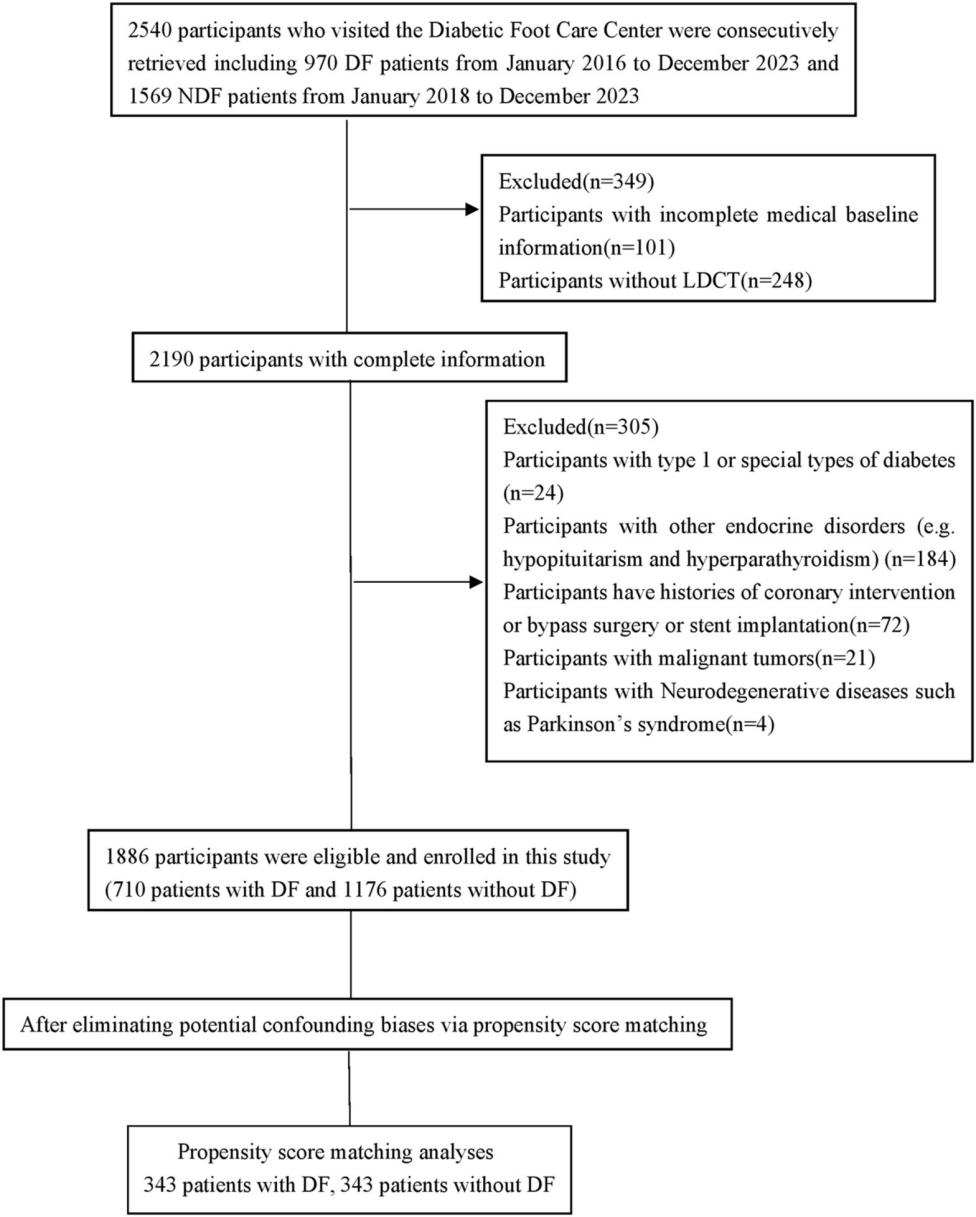
Flowchart illustrating the study selection process. DF, diabetic foot; LDCT, low‐dose computed tomography; NDF, nondiabetic foot.

The retrospective study was reviewed and approved by the Institutional Ethics Committee of West China Hospital and has been registered in the Clinical Trial Registry (registration number: CHiCTR2300076628). Given its retrospective nature, the requirement for informed consent was waived. Patients with type 2 diabetes mellitus (T2DM) were consecutively enrolled from January 2018 to December 2023, and individuals with DF were recruited from January 2016 to December 2023. The diagnosis of DM was established in accordance with the criteria specified in the World Health Organization (WHO) report (1999) [18]. DF was defined according to the guidelines published by the International Working Group on the Diabetic Foot (IWGDF) [19]. The exclusion criteria were as follows: (1) incomplete medical documentation; (2) type 1 diabetes or other specific types of diabetes; (3) history of severe cardiovascular disease, including prior coronary interventions, bypass surgeries, or stent implantations, which may compromise the accuracy of CT‐based calcification scoring; (4) concomitant endocrine disorders, such as hypopituitarism, thyroid or parathyroid disorders, primary aldosteronism; (5) other systemic conditions, including fever, active malignancy, or autoimmune diseases such as rheumatoid arthritis or systemic lupus erythematosus; and (6) pregnancy or lactation. After applying these criteria, 1886 patients with T2DM (710 with DF and 1176 without DF) were initially enrolled. Propensity score matching (PSM) of the overall study population minimized potential confounding imbalances, resulting in a balanced analytical sample of 686 T2DM patients (Figure 1).

### 2.2 CT Scanner and Scanning Parameters

All subjects underwent low‐dose chest CT scanning using a Siemens Somatom Emotion Duo dual‐slice spiral CT scanner. The scanning parameters were as follows: a tube voltage of 120 kV, automated tube current modulation, a slice thickness of 5 mm, a pitch factor of 1.2, a scan matrix size of 512 × 512, and a gantry rotation time of 0.5 s. Images were reconstructed using iterative reconstruction algorithms with a standard soft‐tissue convolution kernel. Scans were conducted during maximal inspiration, with standardized breath‐hold instructions provided to ensure optimal image quality.

### 2.3 Assessment of CT Images

The methods for measuring CAC described in the literature based on LDCT include extent‐based grading, the Weston score, and length‐based grading [20]. Given the well‐documented superior sensitivity and specificity of length‐based quantification demonstrated in prior studies [10], this study employed the length‐based scoring approach to assess CAC. The length‐based grading system assigns a score based on the total length of calcified segments in the coronary arteries: 0 (no calcification); 1 (< 3 mm); 2 (3–5 mm); 3 (6–11 mm); 5 (12–25 mm); and 9 (> 25 mm). The calcification length was determined either through direct measurement on axial images or by estimation based on the slice location. The cumulative length score classifies CAC severity into four categories: absent (0), mild (1–3), moderate (4–8), or severe (≥ 9) [20]. CAC assessments were conducted independently by three trained raters in this study of 1886 participants. Prior to the main analysis, a random sample of 50 patients was selected, and all three raters performed independent evaluations of these cases simultaneously. High inter‐rater agreement was observed, as reflected by Kendall's *W* coefficient of concordance (*W* = 0.875, *p* < 0.001), indicating consistent and reliable CAC scoring throughout the study population.

### 2.4 Assessment of Covariates

Covariates assessed included the duration of diabetes and hypertension, current smoking status, alcohol consumption, as well as comorbidities and complications associated with DM, such as diabetic peripheral neuropathy (DPN), diabetic retinopathy (DR), hypertension (HBP), CAD, ischemic cerebrovascular disease (CVD), and peripheral artery disease (PAD). The diagnosis of DPN was determined based on the presence of neuropathic symptoms, clinical signs, supported by abnormalities detected through neurophysiologic testing [21]. DR was diagnosed by identifying characteristic retinal lesions via fundoscopy or optical coherence tomography, including microaneurysms, hemorrhages, exudates, or diabetic macular edema [22]. HBP was defined in accordance with the 2018 Guidelines as a systolic blood pressure (SBP) ≥ 140 mmHg and/or diastolic blood pressure (DBP) values ≥ 90 mmHg [23]. CAD was diagnosed based on documented evidence of angina, a history of myocardial infarction, acute myocardial infarction at the time of assessment, or coronary artery stenosis with luminal narrowing exceeding 50%. Ischemic CVD was defined as cerebral tissue degeneration, necrosis, or transient functional impairment resulting from cerebral arterial stenosis or occlusion [24]. PAD was diagnosed based on a history of revascularization, the presence of clinical symptoms or signs (e.g., claudication, or absent peripheral pulses), or objective diagnostic criteria, such as an ankle‐brachial index (ABI) ≤ 0.9, arterial stenosis with a diameter reduction of more than 50%, or confirmed arterial occlusion as assessed by lower‐extremity color Doppler ultrasound or angiography [25]. Body mass index (BMI) was calculated as weight in kilograms divided by the square of height in meters [26]. Glycated hemoglobin (HbA1c), triglycerides (TG), total cholesterol (TC), high‐density lipoprotein cholesterol (HDL‐C), low‐density lipoprotein cholesterol (LDL‐C), serum creatinine, and albumin‐to‐creatinine ratio (ACR) were measured at the central laboratory of West China Hospital. Estimated glomerular filtration rate (eGFR) was calculated using standardized equations incorporating age, sex, and calibrated serum creatinine values.

### 2.5 Statistical Analysis

Statistical analyses were carried out using SAS 9.4 software (SAS Institute Inc., Cary, NC, USA). Continuous variables were expressed as mean ± standard deviation or medians with interquartile ranges and were compared using the Student's *t*‐test or the Wilcoxon rank‐sum test based on distribution. Categorical variables were presented as frequencies and percentages, and inter‐group differences were assessed using the *χ* ^2^ test or Fisher's exact test. For each independent variable, tolerance and the variance inflation factor were calculated to evaluate potential multicollinearity. The proportional odds assumption was examined via the parallel lines test to ensure the consistency of slope coefficients across response categories. Multivariable logistic regression models were applied to estimate adjusted odds ratios and their corresponding 95% confidence intervals, which were used to evaluate the association between DF and the severity of CAC after controlling for potential confounding variables. Additionally, PSM in a 1:1 ratio using the nearest neighbor algorithm with a caliper width of 0.05 was performed using R's MatchIt package (RStudio, PBC, Boston, Massachusetts, USA) to balance confounders, with subsequent regression on the matched dataset. Logistic regression analyses were also carried out after PSM. Stratified analysis was conducted to investigate effect modification. The significance of effect modification was formally tested by including interaction terms in the regression model, and the *p* value for the interaction term was calculated. Statistical significance was accepted as a two‐sided test with an *α* level of 0.05.

## 3 Results

The demographic and clinical characteristics of all study participants are presented in Table 1. Compared with individuals in the non‐DF group, those in the DF group were predominantly male, older in age, and had longer durations of diabetes and hypertension. Furthermore, the DF group demonstrated a higher prevalence of smoking, a lower BMI, reduced HbA1c levels, and decreased levels of TC, TG, and LDL‐C. This group also exhibited lower hemoglobin concentrations, reduced eGFR, elevated ACR, and a higher prevalence of comorbidities such as DR, DPN, PAD, CVD, CAD, and HBP. No statistically significant differences between the two groups were observed in terms of alcohol consumption, blood pressure parameters (SBP, DBP, MBP), HDL‐C, and medication use.

**TABLE 1:** Demographic and clinical characteristics of study participants.

Variables	DF group	Non‐DF group	*p*
No. of patients (*n*)	710	1176	
Sex (male)%	476 (67.04)	716 (60.88)	0.007
Age (years)	65 ± 13	58 ± 15	< 0.001
Duration HBP (years)^a^	5 (0.17, 10)	6 (1, 13)	0.01
Duration DM (years)	12 (7, 20)	10 (4, 17)	< 0.001
Alcohol consumption (%)			0.86
Never	469 (66.2)	775 (66.9)	
Occasional	85 (11.9)	159 (12.8)	
Frequent	156 (21.9)	251 (21.3)	
Current smoking (%)	317 (44.7)	437 (37.2)	0.001
BMI (kg/m^2^)	23.27 ± 3.25	24.30 ± 3.93	< 0.001
SBP (mmHg)	137.0 ± 21.8	137.3 ± 21.2	0.72
DBP (mmHg)	82.2 ± 12.5	82.3 ± 12.5	0.80
MBP (mmHg)	100.4 ± 13.7	100.7 ± 13.4	0.73
HbA1c (%)	8.50 ± 2.13	9.39 ± 2.53	< 0.001
TC (mmol/L)	3.98 ± 1.18	4.57 ± 1.34	< 0.001
TG (mmol/L)	1.35 (1.01, 1.87)	1.53 (1.06, 2.50)	< 0.001
HDL‐C (mmol/L)	1.11 ± 0.40	1.12 ± 0.39	0.63
LDL‐C (mmol/L)	2.29 ± 0.96	2.59 ± 0.96	< 0.001
Hb (g/L)	112.2 ± 23.5	133.0 ± 21.4	< 0.001
ACR (mg/g)	172.05 (27.3, 921.4)	45.50 (8.0, 732.8)	< 0.001
eGFR (mL/min/1.73 m^2^)	72.50 ± 31.49	86.31 ± 27.52	< 0.001
Complications and comorbidities			
DR	328 (44.7)	251 (21.3)	< 0.001
DPN	661 (93.1)	627 (53.3)	< 0.001
PAD	342 (48.2)	86 (7.3)	< 0.001
HBP	466 (65.6)	673 (57.2)	< 0.001
CVD	110 (15.5)	100 (8.5)	< 0.001
CAD	154 (21.7)	109 (9.3)	< 0.001
Medication information^b^			
Metformin	407 (63.5)	699 (59.9)	0.13
SGLT‐2i	127 (19.8)	220 (18.9)	0.62
GLP‐1RA	13 (2.0)	51 (4.4)	0.01
DPP‐4i	85 (13.3)	157 (13.5)	0.91
Statins	205 (32.0)	337 (28.9)	0.17

*Note:* Data are expressed as mean (SD), median (IQR), or *n* (percentage), as appropriate.

Abbreviations: ACR, albumin‐to‐creatinine ratio; BMI, body mass index; CAD, coronary artery disease; CVD, cerebrovascular disease; DBP, diastolic blood pressure; DF, diabetic foot; DM, diabetes mellitus; DPN, diabetic peripheral neuropathy; DPP‐4i, dipeptidyl peptidase‐4 inhibitors; DR, diabetic retinopathy; eGFR, estimated glomerular filtration rate; GLP‐1RA, glucagon‐like peptide‐1 receptor agonists; Hb, hemoglobin; HbA1c, glycated hemoglobin; HBP, hypertension; HDL‐C, high‐density lipoprotein cholesterol; LDL‐C, low‐density lipoprotein cholesterol; MBP, mean blood pressure; PAD, peripheral arterial disease; SBP, systolic blood pressure; SGLT‐2i, sodium‐glucose cotransporter 2 inhibitors; TC, total cholesterol; TG, triglycerides.

^a^ Duration of HBP was determined by excluding patients without hypertension, followed by a comparison between 624 non‐DF patients and 416 patients with DF.

^b^ Regarding medication information, data from 641 patients with DF and 1167 patients without DF were analyzed after excluding 78 individuals with incomplete medication data.

**FIGURE 2:**
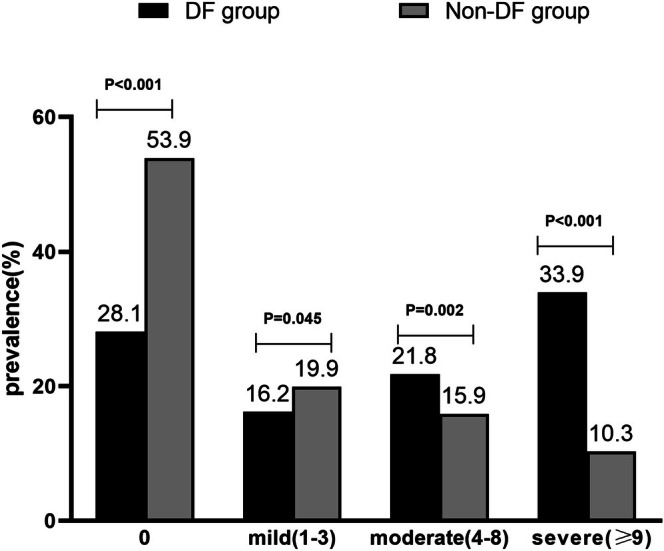
The prevalence and severity of coronary artery calcification in participants with and without diabetic foot. DF, diabetic foot.

Utilizing the length‐based grading approach, both the prevalence and severity of CAC were found to be significantly greater in individuals with DF compared to those without DF (all *p* < 0.05). Among non‐DF patients, the prevalence of CAC was 46.1%, with mild, moderate, and severe calcification observed in 19.9%, 15.9%, and 10.3% of cases, respectively. In contrast, the DF group exhibited a significantly higher calcification prevalence of CAC at 71.9%, along with a more pronounced distribution of severity: 16.2% mild, 21.8% moderate, and 33.9% severe. Notably, individuals with DF demonstrated a 3.3‐fold higher proportion of severe calcification compared to those without DF (33.9% vs. 10.3%, *p* < 0.001), as shown in Figure 2.

The multivariable logistic regression analyses showed the association between DF and the severity of CAC, an association that remained statistically significant across all five models (*p* < 0.001) after stepwise adjustment for potential confounding factors, with individuals diagnosed with DF consistently exhibiting significantly higher levels of coronary calcification compared to controls. Additionally, advanced age, longer duration of hypertension and diabetes, presence of CAD, elevated HDL‐C levels, and comorbidities including DR, PAD, and DPN were independently associated with increased severity of CAC (Table 2).

**TABLE 2:** Multivariable logistic regression analysis showing the association between diabetic foot and coronary artery calcification across five sequential models.

Variables	Model 1		Model 2		Model 3		Model 4		Model 5	
	OR (95% CI)	*p*	OR (95% CI)	*p*	OR (95% CI)	*p*	OR (95% CI)	*p*	OR (95% CI)	*p*
DF	2.61 (2.17, 3.13)	< 0.001	1.60 (1.27, 2.01)	< 0.001	1.96 (1.49, 2.57)	< 0.001	1.53 (1.18, 1.99)	0.001	1.79 (1.32, 2.43)	< 0.001
Age	1.07 (1.06, 1.08)	< 0.001	1.04 (1.04, 1.05)	< 0.001	1.05 (1.03, 1.06)	< 0.001	1.05 (1.04, 1.06)	< 0.001	1.05 (1.03, 1.06)	< 0.001
Duration_HBP	—	—	1.04 (1.03, 1.05)	< 0.001	1.05 (1.03, 1.06)	< 0.001	1.05 (1.03, 1.07)	< 0.001	1.06 (1.04, 1.08)	< 0.001
Duration_DM	—	—	1.03 (1.02, 1.05)	< 0.001	1.03 (1.02, 1.05)	< 0.001	1.03 (1.02, 1.04)	< 0.001	1.03 (1.01, 1.04)	< 0.001
Drinking	—	—	1.40 (1.07, 1.82)	0.014	1.39 (1.02, 1.91)	0.040	1.49 (1.09, 2.03)	0.012	1.44 (1.01, 2.07)	0.044
HDL	—	—	1.48 (1.18, 1.85)	< 0.001	1.60 (1.23, 2.08)	< 0.001	1.48 (1.15, 1.90)	0.002	1.53 (1.15, 2.04)	0.003
CAD	—	—	2.00 (1.54, 2.60)	< 0.001	2.55 (1.84, 3.55)	< 0.001	2.28 (1.63, 3.18)	< 0.001	2.87 (1.91, 4.32)	< 0.001
DR	—	—	1.45 (1.18, 1.79)	< 0.001	1.43 (1.12, 1.84)	0.005	1.53 (1.19, 1.96)	< 0.001	1.41 (1.05, 1.89)	0.022
PAD	—	—	2.61 (2.03, 3.34)	< 0.001	2.25 (1.66, 3.04)	< 0.001	—	—	—	—
DPN	—	—	1.26 (1.01, 1.58)	0.042	—	—	1.37 (1.07, 1.76)	0.011	1.37 (1.04, 1.81)	0.026

*Note:* The reference categories for variables are as follows: Drinking (no), CAD (no), DR (no), PAD (no), and DPN (no). The outcome variable was the ordinal CAC severity grade (0 = none, 1 = mild, 2 = moderate, 3 = severe). Odds ratios (ORs) from ordinal logistic regression represent the odds of being in a higher CAC severity category (e.g., severe vs. moderate/mild/none) for a one‐unit change in the predictor variable.

Model 1: Adjusted for sex and age.

Model 2: Adjusted for sex, age, duration of diabetes and hypertension, BMI, smoking history, alcohol consumption, HbA1c, MBP, eGFR, ACR, CAD, CVD, PAD, DR, DPN, TC, LDL, and HDL.

Model 3: Adjusted for the same variables as Model 2, with exclusion of patients having an eGFR < 60 mL/min/1.73 m^2^.

Model 4: Adjusted for the same variables as Model 2, with exclusion of patients with a confirmed diagnosis of PAD.

Model 5: Adjusted for the same variables as Model 2, with exclusion of patients who have both an eGFR < 60 mL/min/1.73 m^2^ and a confirmed diagnosis of PAD.

Abbreviations: ACR, albumin‐to‐creatinine ratio; BMI, body mass index; CAD, coronary artery disease; CI, confidence interval; CVD, cerebrovascular disease; DPN, diabetic peripheral neuropathy; DR, diabetic retinopathy; eGFR, estimated glomerular filtration rate; HbA1c, hemoglobin A1c; HDL, high‐density lipoprotein; LDL, low‐density lipoprotein; MBP, mean blood pressure; OR, odds ratio; PAD, peripheral artery disease; TC, total cholesterol.

**FIGURE 3:**
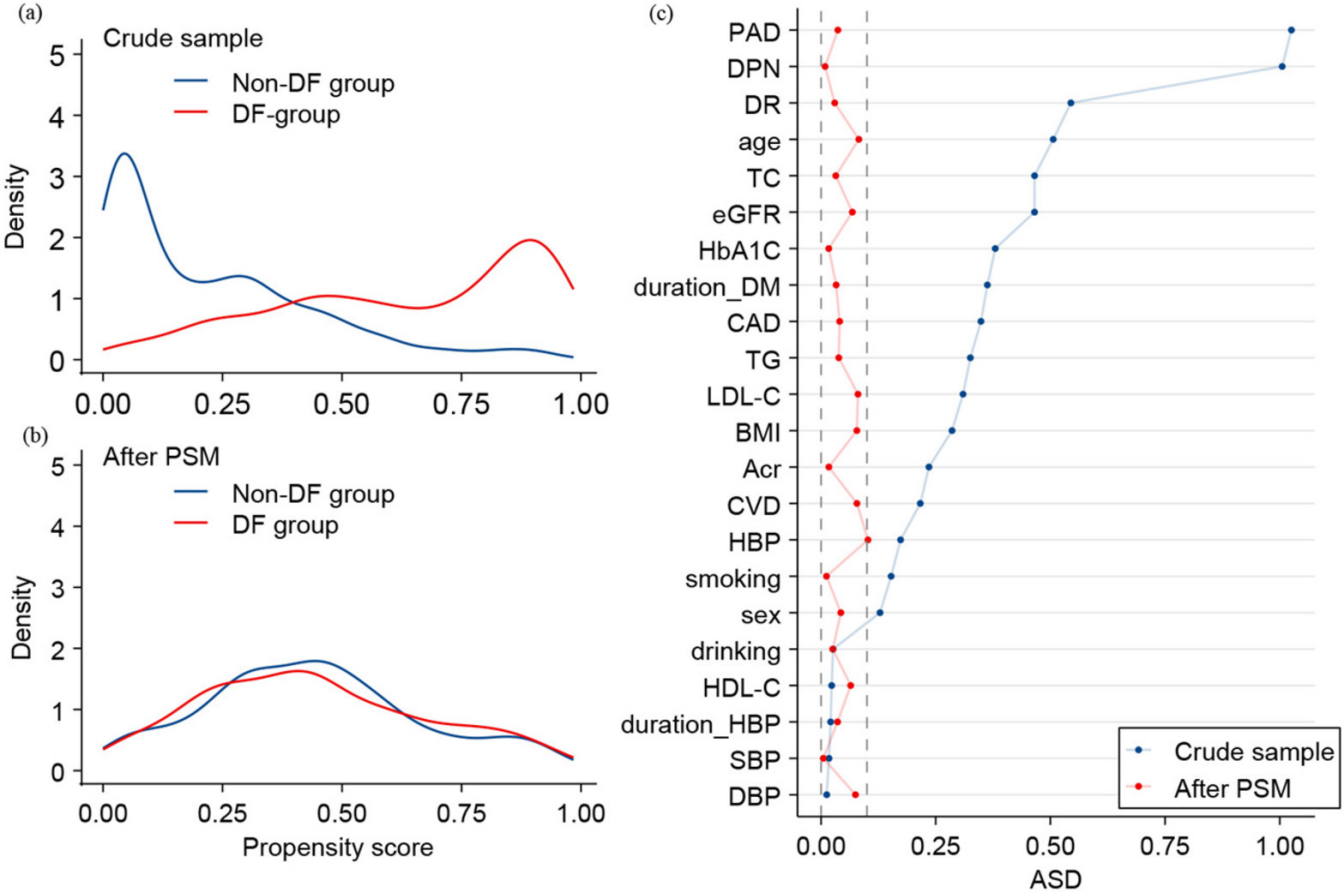
Propensity scores (PS) distributional overlap and absolute standardized differences (ASD) in participants with and without diabetic foot (DF). (a and b) Show the PS distributions between participants with and without DF in the crude sample and the PSM‐matched sample, respectively. The area under the probability density curve along the x‐axis represents the probability of corresponding PS values, with smoothing performed via kernel density estimation. Greater overlap of the two PS curves indicates lower confounding risk. (c) Dot plot of absolute standardized mean differences for all covariates before and after matching. The dashed line denotes the 0.1 imbalance threshold, a widely used metric for notable between‐group covariate imbalance. ACR, albumin‐to‐creatinine ratio; ASD, absolute standardized differences; BMI, body mass index; CAD, coronary artery disease; CVD, cerebrovascular disease; DBP, diastolic blood pressure; DF, diabetic foot; DM, diabetes mellitus; DPN, diabetic peripheral neuropathy; DR, diabetic retinopathy; eGFR, estimated glomerular filtration rate; HbA1c, hemoglobin A1c; HBP, hypertension; HDL, high‐density lipoprotein; LDL, low‐density lipoprotein; MBP, mean blood pressure; PAD, peripheral artery disease; PSM, propensity score matching; SBP, systolic blood pressure; TC, total cholesterol; TG, triglycerides.

To better balance covariates between the two groups, PSM was employed to validate the association between DF and the severity of CAC. Figure 3a,b illustrates the distribution of PS among individuals with and without DF, both before and after PSM. Improved overlap in the PS distributions between the two groups indicates a reduction in potential confounding bias. Figure 3c presents a dot plot of the absolute standardized differences for covariates before and after matching. The dashed line represents the threshold for substantial imbalance, defined as an absolute standardized difference greater than 0.1. In the PSM analysis, the results consistently demonstrated that patients with DF exhibited significantly higher levels of calcification compared to those without DF (OR 1.72; 95% CI [1.28–2.32]; *p* < 0.001) (Table 3).

**TABLE 3:** Multivariable logistic regression for coronary artery calcification after propensity score matching.

Variables	OR, 95% CI	*p*
DF	1.72 (1.28, 2.32)	< 0.001
Age	1.06 (1.04, 1.07)	< 0.001
Duration_HBP	1.04 (1.02, 1.06)	< 0.001
Duration_DM	1.02 (1.00, 1.04)	0.024
eGFR	1.00 (0.99, 1.00)	0.18
MBP	0.99 (0.98, 1.00)	0.060
CAD	2.41 (1.58, 3.69)	< 0.001
DR	2.00 (1.46, 2.76)	< 0.001
PAD	2.34 (1.57, 3.49)	< 0.001
Drinking (frequent)	2.00 (1.33, 3.02)	< 0.001

*Note:* Data analysis was conducted following PSM, with adjustments made for the following covariates: sex, age, duration of diabetes and hypertension, BMI, history of smoking and alcohol consumption, HbA1c, MBP, eGFR, ACR, CAD, CVD, PAD, DR, DPN, TC, LDL, and HDL.

Abbreviations: ACR, albumin‐to‐creatinine ratio; BMI, body mass index; CAD, coronary artery disease; CVD, cerebrovascular disease; DPN, diabetic peripheral neuropathy; DR, diabetic retinopathy; eGFR, estimated glomerular filtration rate; HbA1c, glycated hemoglobin A1c; HBP, hypertension; HDL, high‐density lipoprotein; LDL, low‐density lipoprotein; MBP, mean blood pressure; PAD, peripheral artery disease; PSM, propensity score matching; TC, total cholesterol.

**FIGURE 4:**
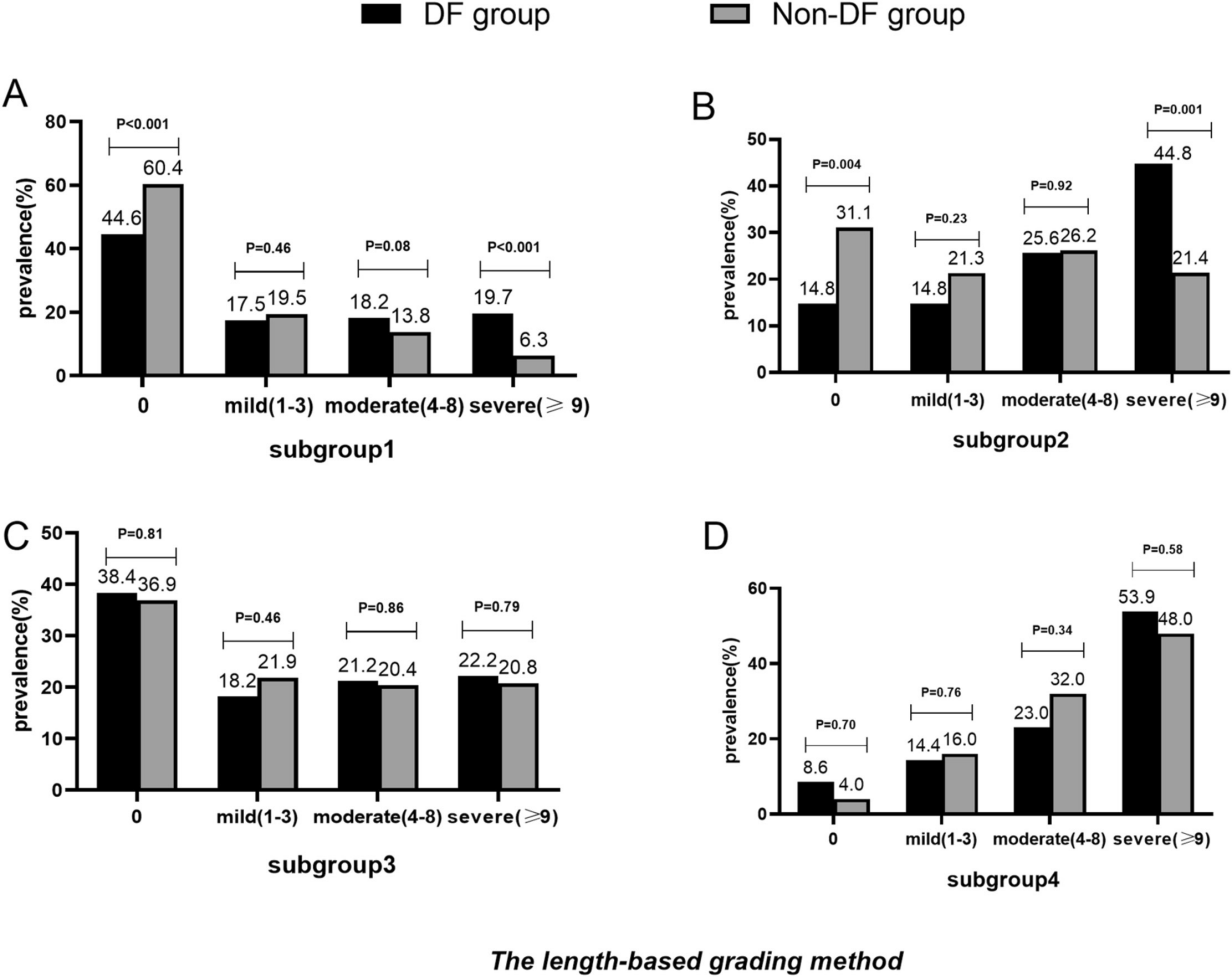
The prevalence and severity of coronary artery calcification across four subgroups. *Note:* (A) Subgroup 1 consisted of participants without PAD and an eGFR ≥ 60 mL/min/1.73 m^2^; (B) Subgroup 2 included those with PAD and an eGFR ≥ 60 mL/min/1.73 m^2^; (C) Subgroup 3 comprised individuals without PAD and an eGFR < 60 mL/min/1.73 m^2^; and (D) Subgroup 4 consisted of participants with PAD and an eGFR < 60 mL/min/1.73 m^2^. DF, diabetic foot.

Based on PAD status and renal function (eGFR ≥ 60 or < 60 mL/min/1.73 m^2^), the study population was divided into four subgroups. Subgroup 1 consisted of participants without PAD and an eGFR ≥ 60 mL/min/1.73 m^2^; Subgroup 2 included those with PAD and an eGFR ≥ 60 mL/min/1.73 m^2^; Subgroup 3 comprised individuals without PAD and an eGFR < 60 mL/min/1.73 m^2^; and Subgroup 4 consisted of participants with PAD and an eGFR < 60 mL/min/1.73 m^2^. Significant differences in severe CAC prevalence between DF and non‐DF groups were found in patients with preserved renal function, both without PAD (Subgroup 1, *p* < 0.001) and with PAD (Subgroup 2, *p* = 0.001). In contrast, no significant differences were observed in those with renal impairment, regardless of PAD status (Subgroups 3 and 4, *p* = 0.79 and *p* = 0.58, respectively; Figure 4).

**FIGURE 5:**
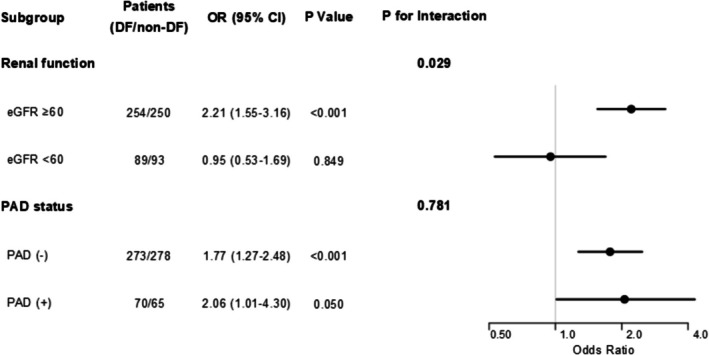
Stratified analysis of the association between diabetic foot and coronary artery calcification. CI, confidence interval; DF, diabetic foot; eGFR, estimated glomerular filtration rate; OR, odds ratio; PAD, peripheral artery disease.

To investigate the potential effect modification of renal function and PAD status on the association between DF and CAC, stratified analyses alongside interaction tests were conducted on the population after PSM. Results revealed a statistically significant interaction between DF and renal function (*p* for interaction = 0.029). Among patients with preserved renal function, DF was significantly associated with CAC (OR = 2.21, 95% CI [1.55–3.16], *p* < 0.001). Conversely, in patients with renal impairment, this association was completely attenuated and non‐significant (OR 0.95, 95% CI [0.53–1.69], *p* = 0.849). No statistically significant interaction was observed between DF and PAD status (*p* for interaction = 0.781). The association between DF and CAC remained consistent across different PAD statuses (Figure 5).

## 4 Discussion

This large‐scale cross‐sectional study of patients with T2DM revealed a significantly higher prevalence of CAC among individuals with DF compared to those without DF, as evaluated using a length‐based visual scoring method on LDCT. This association was particularly pronounced in patients with preserved renal function (eGFR ≥ 60 mL/min/1.73 m^2^), where the DF group demonstrated substantially more severe CAC irrespective of PAD status. In contrast, among patients with impaired renal function (eGFR < 60 mL/min/1.73 m^2^), the severity of CAC was similar between the DF and non‐DF groups, with levels in both substantially elevated compared to their counterparts with preserved renal function. Crucially, DF was independently associated with CAC after comprehensive adjustment for demographic, lifestyle, and clinical confounders. These findings remained statistically robust following sensitivity analyses that excluded participants based on renal function and PAD status, as well as after additional adjustment through PSM.

Previous studies, predominantly using the Agaston scoring system, have established a higher prevalence and severity of CAC in diabetic than in nondiabetic populations [27, 28]. While the overall prevalence of any CAC in our research (55.8%) was somewhat lower than in some Agatston‐based studies, this may be attributable to the known lower sensitivity of visual assessment for detecting microcalcifications [29]. However, the prevalence of severe CAC in our entire study population (19.2%) and particularly in the DF group (33.9%) was more substantial than prior research [28]. This discrepancy in severe CAC prevalence may be partly explained by the fact that severe calcification in T2DM often presents as diffuse, sheet‐like deposits [30]. A length‐based visual assessment on LDCT may effectively capture this diffuse, longitudinal calcification burden, whereas the Agatston score primarily emphasizes calcification density and area [31]. Nevertheless, further prospective studies are needed to determine whether the LDCT‐based visual length assessment is superior to the Agatston score in predicting long‐term cardiovascular adverse events and mortality among patients with DF.

This study focused on severe CAC as the primary endpoint because this degree of calcification burden, as defined by length‐based method, has been clearly demonstrated to be strongly associated with an increased risk of major adverse cardiovascular events [32]. Building upon this, our study revealed the prevalence of severe CAC in the DF group was 33.9%, approximately three times higher than that in the non‐DF group (10.3%), and there was a strong link between DF and the severity of CAC in patients with preserved renal function. This finding positions DF as a potential marker of high cardiovascular risk in this population, warranting early CAC screening.

The pathophysiological link between DF and accelerated CAC is likely multifactorial, driven not solely by traditional risk factors such as hyperglycemia but also by distinct pathological mechanisms specifically associated with DF. We propose that these specific mechanisms are most fully manifested in the absence of advanced kidney disease, consistent with our observed stronger association in patients with preserved renal function. Chronic ulcers release necrotic debris and pathogen‐associated molecular patterns, activating the innate immune system and elevating pro‐inflammatory cytokines, including IL‐1β, IL‐6, and TNF‐α, which promote vascular inflammation and calcification [33, 34]. Repeated surgical debridement may exacerbate systemic inflammatory responses, potentially accelerating coronary calcification [35]. Additionally, the persistent hyperglycemic wound environment, in conjunction with chronic inflammation, facilitates the accumulation of advanced glycation end products (AGEs). The interaction between AGEs and their receptor induces oxidative stress and promotes the osteogenic transformation of vascular smooth muscle cells, thereby enhancing calcium deposition in the vasculature [36, 37]. Associated neurovascular complications may contribute to endothelial dysfunction [38], while comorbidities such as vitamin D deficiency can disrupt calcium–phosphate homeostasis and promote vascular calcification [39]. Although these pathways provide partial insights, the complete pathophysiological mechanism remains to be further elucidated.

In addition, renal function status emerged as a key factor modulating the relationship between DF and CAC. Among patients with impaired renal function, no statistically significant difference in the prevalence of severe CAC was observed between individuals with DF and those without DF, a finding confirmed by a significant statistical interaction (*p* for interaction = 0.029). Concurrently, CAC levels were higher in patients with impaired renal function compared to those with normal renal function, irrespective of DF status, reinforcing that special pathological mechanisms of the kidney become the primary driver of vascular calcification in advanced kidney disease, which may overshadow the cardiovascular risk attributable to DF itself [40]. Therefore, the assessment and management of vascular calcification is crucial in patients with impaired renal function, irrespective of DF status.

This study has several strengths, including its focus on a high‐risk DF population, a relatively large sample size enabling robust PSM, and the comprehensive collection of medication and comorbidity data. To the best of our knowledge, no previous investigations have simultaneously controlled for or adjusted multiple key confounding factors, especially PAD, DPN, DR, CAD, CVD, duration of diabetes, and age, with comparable rigor. Beyond this, our analysis progressed beyond establishing basic associations, further exploring effect modification through stratification and formal interaction tests.

Nevertheless, several limitations warrant consideration. Firstly, the cross‐sectional design precludes causal inference, highlighting the necessity for longitudinal follow‐up studies to establish temporal relationships. Secondly, the single‐center nature of the study may introduce selection bias, thereby emphasizing the need for future multicenter investigations to validate these findings in more diverse and representative populations. Despite these limitations, the rigorous adjustment for potential confounding factors enhances the reliability and validity of the observed findings. Finally, although individuals with DF demonstrated greater calcification severity, the clinical implications of this difference, particularly with respect to cardiovascular mortality risk, remain uncertain. To address this knowledge gap, an ongoing prospective cohort study is currently underway to further investigate this association.

## 5 Conclusion

In conclusion, the present study demonstrates that DF is independently associated with the severity of CAC in individuals with T2DM. Among individuals with preserved renal function, DF was independently associated with the severity of CAC, irrespective of the presence of concomitant PAD. In contrast, among those with impaired renal function, no significant differences were observed in the prevalence of mild, moderate, or severe CAC between patients with DF and without DF; however, the overall burden of CAC in this population was markedly higher compared to those with preserved renal function. These findings emphasize the importance of enhanced surveillance for coronary calcification in individuals with either impaired renal function or DF, particularly in the context of preserved renal function. Future prospective studies are warranted to investigate whether increased CAC severity contributes to an elevated risk of cardiovascular mortality in DF patients.

## Author Contributions

**Xingwu Ran**, **Yun Gao** and **Chunchao Xia:** study conception and design. **Chunchao Xia**, **Feiyan Shi**, **Mingxin Bai**, **Ruixue Feng**, **Chunjie Ma** and **Donge Yan:** data collection and analysis. **Yun Gao**, **Feiyan Shi** and **Sen He:** statistical analysis. **Feiyan Shi and Yun Gao:** manuscript preparation. **Xingwu Ran**, **Chunchao Xia**, **Yun Gao**, **Dawei Chen**, **Chun Wang**, **Lihong Chen**, **Donge Yan** and **Sen He:** manuscript review and editing. All authors reviewed and approved the final version of the manuscript.

## Funding

This work was supported by Sichuan Science and Technology Program, 2024YFFK0290; Health Commission of Sichuan Province Medical Science and Technology Program, 24LCYJZD02; Health Commission of Sichuan Province, 23LCYJ042; 1.3.5 Project for Disciplines of Excellence, West China Hospital of Sichuan University, ZYGD24005; 1.3.5 Project of Center for High Altitude Medicine, West China Hospital, Sichuan University, GYYX24002; and Sichuan Medical Association Special Research Project, HX‐H2502066.

## Ethics Statement

The study was reviewed and approved by the Institutional Ethics Committee of West China Hospital and has been registered in the Clinical Trial Registry (registration number: CHiCTR2300076628).

## Consent

Given its retrospective nature, the requirement for informed consent was waived by the West China Hospital.

## Conflicts of Interest

The authors declare no conflicts of interest.

## Data Availability

The data that support the findings of this study are available from the corresponding author upon reasonable request.
